# Regulatory T‐cell heterogeneity

**DOI:** 10.1002/cti2.1012

**Published:** 2018-03-29

**Authors:** Kirsten A Ward‐Hartstonge, Ajithkumar Vasanthakumar

**Affiliations:** ^1^ Microbiology and Immunology Department University of Otago Dunedin New Zealand; ^2^ The Peter Doherty Institute for Infection and Immunity University of Melbourne Melbourne VIC Australia

Regulatory T cells (Tregs) preserve immune homeostasis by suppressing autoimmunity and inflammation. While most Tregs develop in the thymus, they are heterogenous in their tissue localisation and function. This heterogeneity is advantageous for the maintenance of immune and tissue homeostasis in organs such as gut and kidney but often detrimental in the context of tumor, since Treg cells can dampen antitumor responses to impact patient survival. Therefore, understanding mechanisms that underlie Treg cell heterogeneity is critical for the effective treatment of diseases that affect specific tissues and organs. In this Special Feature of *Clinical & Translational Immunology,* experts discuss the heterogeneity of regulatory T cells and their role in health and disease.

Ward‐Hartstonge and Kemp^1^ begin this Special Feature by reviewing the heterogeneity of Tregs present in human cancers with a specific focus on how these Treg subsets impact patient outcomes. Tregs from the tumor microenvironment often have a different phenotype and function compared to those found outside the tumor microenvironment. The tumor microenvironment can alter Treg phenotypes in the tumor and can ultimately impact patient survival. The authors discuss the limitations of using FOXP3 alone to identify Tregs due to the presence of multiple FOXP3^+^ Treg cell subsets and FOXP3^+^ conventional T‐cell populations within human tumors. This review highlights the need for detailed and sophisticated analysis of Tregs in tumor sites in order to identify tumor‐specific Treg cell molecules that can be targeted to treat cancer.

Tregs in the gut establish tolerance to commensal microbial antigens and dietary antigens. Perturbations in gut Treg function, both environmental and genetic, may be involved in diseases such as inflammatory bowel diseases and coeliac disease. In this Special Feature, Luu *et al*.^2^ discuss the developmental dichotomy of gut Tregs and discuss mechanisms that underpin these developmental pathways.

Tregs are critical to suppress renal inflammation. Alikhan *et al*.^3^ discuss how Tregs play a protective role in chronic and acute kidney damage. Tregs also prevent graft versus host disease and promote tolerance in kidney transplantation settings. Tregs that are of a distinct phenotype have been linked to successful kidney transplants suggesting further understanding of kidney resident Tregs might be beneficial to treat kidney inflammation and tolerate kidney transplants.

Mohr *et al*.^4^ describe how human Tregs can be phenotypically, functionally and epigenetically heterogenous and outline how this knowledge can be utilised to restore immune homeostasis or promote tolerance using Tregs.

In the final review of this Special Feature, Sadlon *et al*.^5^ describe several molecular determinants including microRNAs, noncoding RNAs and unique enhancer elements that play critical role in Treg cell development. Extrinsic cues such as inflammation and polymorphisms can perturb these regulatory modules, leading to autoimmunity. This area of study in humans is at its infancy and requires cutting‐edge genomic tools to understand mechanisms that instruct heterogeneity.

Together, these reviews highlight diversity in Treg cell development, tissue localisation and function and discuss mechanisms that underpin this heterogeneity. While examples to demonstrate heterogeneity in Tregs is mounting, exactly how Tregs are licensed to perform a specific function or to populate a specific tissue is still unclear. Understanding the Treg intrinsic and extrinsic mechanisms that license Tregs to adapt to particular tissue or inflammatory milieu will enable treatment of several inflammatory and autoimmune diseases.

## Conflict of interest

The authors declare no conflict of interest.

## References

[1] Ward‐Hartstonge KA , Kemp RA . Regulatory T‐cell heterogeneity and the cancer immune response. Clin Transl Immunol 2017; 6: e154. 10.1038/cti.2017.43 PMC5628269 28983402

[2] Luu M , Steinhoff U , Visekruna A . Functional heterogeneity of gut‐resident regulatory T cells. Clin Transl Immunol 2017; 6: e156. 10.1038/cti.2017.39 PMC5628268 28983404

[3] Alikhan MA , Huynh M , Kitching AR , Ooi JD . Regulatory T cells in renal disease. Clin Transl Immunol 2018; 7: e1004. 10.1002/cti2.1004 PMC5822411 29484182

[4] Mohr A , Malhotra R , Mayer G , Gorochov G , Miyara M . Human FOXP3+ T regulatory cell heterogeneity. Clin Transl Immunol 2018; 7: e1005. 10.1002/cti2.1005 PMC5822410 29484183

[5] Sadlon T , Brown CY , Bandara V *et al* Unravelling the molecular basis for regulatory T‐cell plasticity and loss of function in disease. Clin Transl Immunol 2018; 7: e1011. 10.1002/cti2.1011 PMC5827651 29497530

